# EMPIRICUS micafungin versus placebo during nosocomial sepsis in Candida multi-colonized ICU patients with multiple organ failures: study protocol for a randomized controlled trial

**DOI:** 10.1186/1745-6215-14-399

**Published:** 2013-11-21

**Authors:** Jean-François Timsit, Elie Azoulay, Muriel Cornet, Jean-Pierre Gangneux, Vincent Jullien, Aurélien Vésin, Edith Schir, Michel Wolff

**Affiliations:** 1 University Grenoble 1, Intensive Care Unit, Albert Michallon Hospital, BP 217, 38043 Grenoble, Cedex 9, France; 2 Intensive Care Unit, Saint Louis Hospital, Paris, France; 3 TIMC-IMAG TheREx, UMR 5525 CNR-Joseph Fourier University, Grenoble 1, Parasitology-Mycology, Albert Michallon Hospital, BP 217, 38043 Grenoble, Cedex 9, France; 4 Rennes Teaching Hospital, Parasitology-Mycology Laboratory, University Rennes 1, IRSET INSERM U1085, 35043 Rennes, Cedex 9, France; 5 INSERM U663; Paris Descartes University, Department of Clinical Pharmacology, Georges Pompidou European Hospital; Assistance Publique-Hôpitaux de Paris, Paris, France; 6 University Grenoble 1, U 823, Equipe 11 Outcome of mechanically ventilated patients and airway cancers, Paris, France; 7 Outcomerea Organization, Paris, France; 8 Pharmacology University Grenoble 1, Albert Michallon Hospital, BP 217, 38043 Grenoble, Cedex 9, France; 9 AP-HP, Hôpital Bichat, Medical and Infectious Diseases Intensive Care Unit,F-75018 Paris, France

**Keywords:** Colonization, Nosocomial sepsis, Candidemia, Invasive candidiasis, Micafungin

## Abstract

**Background:**

The potential interest of antifungal treatment of non-immunocompromized patients with sepsis, extra-digestive *Candida* colonization and multiple organ failure is unknown. It represents three-quarters of antifungals prescribed in Intensive Care Units. It may allow early treatment of invasive fungal infection in the incubation phase but expose patients to unnecessary antifungal treatments with subsequent cost and fungal selection pressure. As early diagnostic tests for invasive candidiasis are still considered to be insufficient, the potential interest in this strategy needs to be demonstrated.

**Methods:**

This prospective multicenter, double blind, randomized-controlled trial is conducted in 23 French Intensive Care Units. All adult patients, mechanically ventilated for more than four days with sepsis of unknown origin and with at least one extradigestive fungal colonization site and multiple organ failure are eligible for randomization. Patients with proven invasive candidiasis are not included. After a complete mycological screening, patients are allocated to receive micafungin 100 mg intravenously once a day or placebo for 14 days. We plan to enroll 260 patients. The main objective is to demonstrate that micafungin increases survival of patients without invasive candidiasis at day 28 as compared to placebo. Other outcomes include day 28 and 90 survival and organ failure evolution. Additionally, pharmacokinetics of micafungin in enrolled patients will be measured and evolution of fungal biomarkers and susceptibility profiles of infecting fungi will also be followed.

**Discussion:**

This study will help to provide guidelines for treating non-immunocompromized patients with fungal colonization multiple organ failure and sepsis of unknown origin.

**Trial registration:**

Clinicaltrials.gov number NCT01773876

## Background

*Candida*, one of the most frequently recovered pathogens in patients with hospital acquired bloodstream infections [1,2], is associated with a large increase in mortality [3-5]. *Candida* accounts for up to 17% of all ICU-acquired infections [6] and multiple-site *Candida* colonization is found in approximately half of medical and surgical patients [7,8]. Major risk factors for *Candida* colonization include length of ICU stay, use of parenteral nutrition, broad-spectrum and long-term antibiotics, central lines, and abdominal surgery. Importantly, a continuum exists between *Candida* colonization and candidemia [9]. Thus, a colonization index (number of colonized sites/number of sampled sites) > 0.5 is associated with an increased risk of candidemia with recovery of the same *Candida* species or genotypes in the colonized sites and bloodstream [8]. Studies done to develop a *Candida* score showed that factors associated with candidemia were surgery, multiple-site *Candida* colonization, severe sepsis, and parenteral nutrition [10]. Thus, *Candida* colonization, although not unique, is a reliable independent risk factor for candidemia [11-13]. Therefore, early systemic antifungal therapy (SAT) may deserve consideration in ICU patients.

Delays in initiating appropriate treatment have been associated with increased mortality in patients with bloodstream infections [3]. The same findings have been reported in patients with candidemia [14-17]. However, the performance of available diagnostic tools for diagnosing candidemia remains limited [11,12,18,19]. Therefore, in an attempt to decrease *Candida*-related mortality, an increasing number of ICU patients without documented candidemia are receiving empirical SAT [20,21]. Over the last 15 years, several studies have evaluated the potential benefits from SAT in ICU patients overall [22-27] and in the subset of ICU patients having risk factors for candidemia or sepsis of unknown origin [28,29]. Prophylactic SAT has been suggested for the sickest surgical ICU patients, most notably those with peritonitis [30]. However, results from a recently published trial in medical-surgical ICU patients do not support routine SAT in patients with nonresolving sepsis but no *Candida* colonization [29]. No study has been conducted in the same patients with multiple-site *Candida* colonization. The appropriate modalities of use [31] of SAT and the potential benefits ascribable to SAT in ICU patients with sepsis and *Candida* colonization have not been reported. We therefore set up the EMPIRICUS trial to answer this question.

## Methods/Design

EMPIRICUS is a prospective, multicenter, randomized, double-blind, and parallel group study comparing the interest of 14-day empirical micafungin treatment (Mycamine™ Astellas Pharma, Levallois Perret, France 100 mg IV once a day) with placebo, regarding survival without invasive candidiasis during 28 days in adult patients with suspected invasive candidiasis (EudraCT 2011-005451-14).

### Ethics

The study involves 23 ICUs in French hospitals (mainly university affiliated) and was approved by the local Independent Ethic Committee (Comité de Protection des Personnes CPP Sud Est V) on 7 December 2011 and the French Health Authorities (AFSSAPS) on 2 December 2011. The University Hospital of Grenoble is the sponsor of the trial.

Each patient will have given written informed consent concerning the study design and outcomes. If the information could not be delivered to the patient, it will be delivered to his/her relatives.

The study is divided in two consecutive periods for each patient:

● the 14-day treatment period starting the day after the inclusion visit (D0);

● the post-treatment evaluation period from D15 to D90 with an evaluation visit at D21, D28 (end-of-study visit, EOS) and collection of information about long-term survival at three months (during a consultation or by phone contact).

In case of discharge from ICU before having completed the 14-day study treatment period, the patient must receive or have received the study treatment during at least seven days.

All patients who received at least one dose of study treatment will be included in the intent-to-treat analysis.

### Aims

The EMPIRICUS study primarily aims to evaluate the efficacy of early empirical treatment with micafungin in adult ICU patient with suspected invasive candidiasis in increasing proven-invasive-candidiasis-free-survival at day 28.

Secondary objectives are:

● To evaluate the impact of empirical micafungin treatment of patients with possible invasive candidiasis on:

All-cause mortality at D28 (EOS) and D90 (three months post-randomization)

Antifungal-free survival at D28

Organ failure evolution throughout the trial

Mechanical ventilation use during the trial

Colonization index evolution throughout the trial

Serum biomarkers ((1–3)-β-D-glucan level, mannan antigenemia, anti-mannan antibodies, blood *Candida* PCR) evolution throughout the trial

Incidence of ventilator-associated bacterial pneumonia

Pharmacokinetic/pharmacodynamic (PK/PD) profile of micafungin in ICU ventilated patients with sepsis

● To characterize the profile of tolerance of micafungin in ICU ventilated patients with sepsis

### Participants

Patients are eligible for inclusion if they fulfill all the inclusion and non inclusion criteria described in Table 1. Patients are screened by the clinical research monitors and investigators in each center on a daily basis according to patient status and known yeast colonization.

**Table 1:** Inclusion and non inclusion criteria

**Main inclusion criteria**	**•** Age ≥ 18 years
	**•** Hospitalization in ICU ≥ five days (120 hours) before randomization
	**•** Suspected but unproven candidiasis:
	Systemic inflammatory response syndrome (SIRS) manifested by two signs among four (body temperature < 36°C or > 38°C; heart rate > 90/minute; respiratory rate > 20/minute or PaCO_2_ < 32 mmHg; white blood cells > 12,000/mm^3^, < 4,000/mm^3^ or > 10% of circulating immature forms)
	Mechanical ventilation ≥ four days
	Presence of a central vein catheter and/or an arterial line
	Use of broad spectrum antibacterial agents ≥ four days during the last seven days
	**•** Organ failure is defined as a sepsis-related organ failure assessment (SOFA) score ≥ 3
	**•**At least one extra-digestive site of *Candida* sp. colonization (urine, mouth, throat, upper and lower respiratory system, skin folds and drains and postoperative aspiration, and so on); positive samples from rectal swab and/or stool culture are not taken into account although they are collected at randomization visit
	**•** No evidence of bacterial infections that explain the symptoms
	**•** No evidence of invasive fungal infections (positive blood culture, direct examination or positive culture from surgery site, deep biopsy with mycosis) or mould infection according to the criteria of the ‘fungal infection cooperative group of EORTC’ [De Pauw 2008 ]
**Main non inclusion criteria**	**•** Proven invasive infection (positive blood culture with yeast, positive culture from surgery site (only samples taken during surgery or by percutaneous puncture), deep biopsy with mycosis) including aspergillosis requiring antifungal treatment at the time of randomization
	**•** Patients status considered by the investigator to inevitably leading to death or to withdrawal of life support within 48 hours
	**•** Antifungal treatment with an echinocandin > one day or with any other antifungal agent > 72 hours the week preceding the inclusion
	**•** Allergy, hypersensitivity or known intolerance to antifungal echinocandins or to any excipient composing the study drug
	**•** Neutropenia (neutrophil count < 500/mm^3^)
	**•** Previous marrow or organ transplantation
	**•** Recent chemotherapy (since less than six months)
	**•** Ongoing systemic immunosuppressant agents therapy, other than corticosteroids at doses < 2 mg/kg/day of prednisolone or equivalent

If an invasive fungal infection is evidenced by the laboratory tests of baseline samples from a patient who has been included, they are withdrawn from the study and treated according to current IDSA guidelines [31].

Proven *Candida* IFIs are defined according to the modified criteria of EORTC/MSG Fungal Infections Consensus Group 2008:

● Histopathologic, cytopathologic, or direct microscopic examination of a specimen obtained by biopsy or needle aspiration (other than mucous membranes) from a normally sterile site showing yeast cells, true hyphae or pseudohyphae, or

● Recovery of *Candida* by culture of a sample obtained by a sterile procedure (including a freshly placed (< 24 hours previously) drain) from a normally sterile site showing a clinical or radiological abnormality consistent with an infectious disease process, or

● Blood culture that yields *Candida*

### Study management

The Steering Committee is composed of three intensivists (JFT, EA, MW), two mycologists (MC and JPG) and two pharmacists (ES, VJ). JFT, EA, MW are in charge of questions to the investigators, of checking the clinical consistencies of the outcome variables, and of asking for complementary clinical data for classifying patients and events. They will validate before database lock, blindly to the study group, all the outcome variables. Definite validation will require consensus. MC and JPG are in charge of the relationship with mycologists between centers, processes and analyses. VJ is responsible for pharmacokinetics dosage and models. ES is responsible for adverse event collection and declaration.

The Data Safety Monitoring Board (DSMB), composed of five external experts, regularly monitors the safety of the trial and will inform the Steering Committee in case of events putting into question the clinical and biological safety of the study drugs (occurrence of SAEs, publication of results of a similar trial, and so on). When the inclusion of the 50th and 150th patients is achieved, the DSMB will be provided with two statistical analyses of the SAEs reported with micafungin, liver function tests (bilirubin, ALT, AST, alkaline phosphatases, prothrombin time), venous thromboembolic and cardiovascular conditions, invasive candidiasis, deaths and study withdrawals.

The Adjudication Committee composed of the investigators and a mycologist will also adjudicate all suspected or proven candidiasis cases before the unblinding of study data.

The Adjudication Committee will review all the data from dying patients before day 28 and of patients for whom an antifungal treatment will be started.

Micafungin is provided by Astellas Laboratories to the Albert Michallon Hospital University of Grenoble, research pharmacist. Lc2 (Lentilly, France) is the CRO responsible for the preparation and labeling of batches and for providing each site with the necessary number of therapeutic units throughout the study progress. FOVEA (Rueil-Malmaison, France) is in charge of the monitoring of each patient file and Delta Consultant (Eybens, France), which has created the e-CRF, generates the queries, prepare a statistical analysis plan for safety interim analyses and the final primary analysis (primary end-point and secondary clinical end-points). OUTCOMEREA (Paris, France) has created the randomization list and will perform the final statistical analysis.

### Randomization and study treatments

Eligible patients are randomly assigned, in a 1:1 ratio, to receive micafungin 100 mg/day (Mycamine®, Astellas Pharma, Levallois Perret, France) or matched placebo (100 ml NaCl 0.9%), administered intravenously as a one-hour perfusion. Randomization is stratified by centers. They also are randomized between two groups: A and B. Randomization is performed via the website https://empiricus.calystene.fr. After having completed the ‘randomization’ web page, the investigator receives the patient’s number. The pharmaceutical order including the patient’s number is transmitted to the pharmacist who prepares treatment (micafungin or placebo) according to the randomization list.

Reconstitution of bags is performed either by the pharmacist (under extractor hood) or by an external nurse, both in unblinded conditions. Micafungin and placebo solutions provided to the site for infusion to the patient are indistinguishable to ensure that the medication blinding is maintained for both patient and investigator throughout the study. Study drug administration starts immediately after the inclusion visit and lasts 14 days.

Time zero is defined by randomization. Pharmaceutical reconstitution and biological sampling are immediately performed and the study treatment is started just after.

A form is filled by the pharmacist for each preparation. The nurse in charge of the patient fills and signs a nurse form with the times of start and end of infusions. Empty vials and packs are checked by the research pharmacist before destruction for the evaluation of compliance for all the patients.

If the existence of an invasive candidiasis at inclusion is evidenced by the analysis of baseline samples (results of the exams performed before or during the first 48 hours of the study treatment), the study treatment will be stopped and the patient will receive antifungal treatment usually prescribed in the investigational site, but blinding is not broken and the patient remains in the intent-to-treat analysis. During the treatment period, if an invasive candidiasis is evidenced, the study treatment is stopped; the antifungal treatment is started according to the decision of the investigators. Investigators can use micafungin, or alternatively, another antifungal drug according to current guidelines [31]. In this latter case, the patient stops taking the study drug but remains in the study and the investigator performs the end-of-treatment assessments immediately and the end-of-study visit 28 days later.

### Data collection

An e-CRF is used for each patient to collect the data. Assessments are made at the screening period, inclusion (D0) and on days 1 to 7, 9, 11, 14, 21, 28 and 90 (phone contact). Assessments include sepsis-related organ failure assessment (SOFA), monitoring (catheter, tubes and drains, mechanical ventilation procedures, inotropes), candidiasis evaluation (signs, imaging, mycological tests), laboratory tests (hematology, biochemistry, urinalysis, pregnancy test at inclusion only), microbiology (blood cultures, mycology). Biomarker dosages are performed blindly to the study group by the mycology and pharmacokinetic investigators and included in separated files. The results are not given to the clinical investigators. The existence of molecular markers of resistance (FKS1 and any other) is investigated in case of positive blood culture. Adverse events, compliance and concomitant treatments are collected at each visit.

In cases of invasive candidiasis, treatment used and complications detected by the attending physicians are collected. At D90, information about patient survival is collected.

### Laboratory tests and pharmacokinetic sampling

Table 2 summarizes all the laboratory tests performed throughout the study.

**Table 2:** Laboratory tests

**Laboratory test**	**Sampling days**	**Measured parameters**
Hematology (3 ml blood sampling, local laboratory)	D0, every day of first week of treatment, D9, D11, D14 (EOT), D21, D28 (EOS)	Hb, hematocrit, WBC, RBC, platelets
Biochemistry (3 ml blood sampling, local laboratory)	D0, every day of first week of treatment, D9, D11, D14 (EOT), D21, D28 (EOS)	Na, K, Ca, glucose, AST, ALT, alkaline phosphatases, total bilirubin, prothrombin time, albumin, serum creatinine
Procalcitonin (local lab)	D0, D3, D7, D14 (EOT)	
Urinalysis (local lab)	D0, D1, D7, D14 (EOT)	Proteins, creatinine
Blood culture (10 ml blood sampling)	D0, D3, D7, D14 (EOT), D28 (EOS)	Analyses in the local laboratory, completed with further centralized analyses in case of positive results
Mycological follow-up of colonization	D0	Culture of specimens of mouth, throat, upper and lower respiratory tract, skin folds, urine and lower gastro-intestinal tract (rectal swab and feces), and if necessary drains, catheters and postoperative aspiration. In case of positive culture, an *in vitro* sensitivity study (E-test) to pre-defined antifungal agents is performed at inclusion on isolates
		Analyses in the local laboratory, completed with further centralized analyses in case of positive results
Centralized microbiological analyses of blood samples and positive blood cultures (Mycology-Parasitology Departments of Grenoble and Rennes University Hospitals)	D0, D3, D7, D14, D28	Biomarkers: blood PCR *Candida* analysis and (1–3)-β-D-glucan level, mannan antigen, anti-mannan antibodies tests if patient exhibits symptoms of invasive candidiasis
		In case of positive blood culture or deep sites: search for molecular resistance markers (biobank)
PK sampling (5 ml blood sampling)	D0, D1	Three samples following first study drug intake and two following the second intake. Sampling times specific to groups A and B

EOT: end-of-treatment.

EOS: end-of-study (day 28).

Microbiological analyses of blood cultures and colonization sites are performed by local laboratories according to standardized procedures for *in vitro* identification of microorganisms. Briefly, specimens for mycological analyses are cultured on Sabouraud-chloramphenicol or any chromogenic medium, according to the usual procedures of the local mycology laboratory, and incubated at 37°C for at least five days. Species identification is performed using carbohydrate assimilation profile or mass spectrometry where available. The objectives of local mycological analyses are to check for the presence of at least one extra-digestive site of *Candida* sp. colonization before inclusion (inclusion criteria). Blood cultures and sampling of any normally sterile site are immediately collected in case of a new or severe sepsis or a septic shock. Furthermore, blood cultures are systematically performed on days 3, 7, 14 and 28. In case of positive culture for *Candida* from a deep site or blood, the specimen is stored in Sabouraud broth at 4°C or in standard freezing medium with glycerol at −80°C before being sent to the Grenoble University Hospital at site closure. The centralized analyses performed on these invasive isolates will include the identification of molecular markers associated with antifungal resistance. Samples for identification of molecular markers of resistance are collected on dry tubes, centrifuged, decanted in one aliquot of about 2 to 2.5 ml of serum and stored in a serum bank at −20°C.

Dosages of biomarkers (blood PCR *Candida*, (1–3)-β-D-glucan level, mannan antigenemia, anti-mannan antibodies) are performed in the centralized laboratories of Parasitology-Mycology of Rennes and Grenoble University Hospitals. Blood samples for mycology and PK/PD as well as isolated strains of proven or treated *Candida* infections are transferred from the sites to Grenoble each month. The biobank (serum aliquots and strains) is equally dispatched between Grenoble and Rennes laboratories during the study progress.

Measurements of PK parameters (distribution volume, clearances, AUC, Cmax, Cmin, AUC/MIC, Cmax/MIC) are performed centrally in the Pharmacology Department of Georges Pompidou University Hospital, Paris Descartes University (Dr V Jullien). Five blood samples for PK analysis are planned for each patient, three after the first study drug perfusion and two after the second, according to the following sampling scheme: at the end of first perfusion; 15 to 30 minutes after; immediately before the second perfusion and between 1 to 3 hours and 8 to 12 hours later.

### Maintenance of blood sample integrity

For PK studies, samples of 5 ml of whole blood are taken, placed in dry heparin tubes and centrifuged within one hour after collection. Following the separation of plasma into two aliquots in polypropylene tubes, aliquots are frozen at −80°C and sent on dry ice to the laboratory once a month. Micafungin plasma concentration will be determined using an ultra-high pressure liquid chromatographic with fluorimetric detection method that was validated according to European guidelines for the validation of bioanalytical methods (Guideline EMEA/CHMP/EWP/192217/2009). Blood samples used for blood cultures are collected with a fungal specific tube if possible, or with a couple of aerobic/anaerobic bottles.

### Sample size and power

Hypotheses for the sample size calculation were:

● The mortality of patients fulfilling the selection criteria has been estimated between 30% and 37% (OUTCOMEREA French database [32,33])

● The candidemia related mortality in case of early treatment is 12% whereas it is 35% when the treatment is delayed (current practice) [17]

● According to Schuster *et al*. [29], IFIs should be expected to be diagnosed in 7.1% of patients receiving antifungal therapy and 20.8% of those receiving placebo (absolute difference 13.7%)

● Traditional sensitivity diagnostic test (blood cultures, culture of sterile site) of IFI diagnosis is 60%

Then, in the micafungin group, the actual incidence of IFIs can be estimated at 11.8% (7.1%/0.6), the candidemia related mortality at 1.4% (11.8% x 12%) and overall events between 31.4% and 38.4%. In the placebo group, the candidemia related mortality can be estimated at 4.13% (11.8% × 35%), the number of additional IFIs diagnosed after randomization at 13.7% [29] and overall events between 49.4% and 56.4%. This difference of 18% in the proportions of events between both groups is then an acceptable hypothesis. A two-sided log-rank test with an overall sample size of 235 subjects (of which 118 are in micafungin group and 117 are in placebo group) achieves 80% power at a 0.0500 significance level to detect a difference of 0.18 between 0.37 and 0.55 - the proportions surviving in groups micafungin and placebo, respectively [34]. This corresponds to a hazard ratio of 0.6013. These results are based on the assumption that the hazard rates are proportional. It could be assumed that 5% of the enrolled population will be included but not randomized. The absence of follow-up of patients at day 28 is very improbable (withdrawal of informed consent) and can be assumed to be 5%. A total number of 260 patients (130 in each arm) should then be included.

### Statistical and PK analysis

Biostatistics and epidemiology team of OUTCOMEREA is in charge of statistical analysis of the study data and will use SAS 9x (SAS, Inc., Cary, NC, USA) and R (R foundation for statistical computing, Vienna, Austria) softwares.

Group comparisons will be performed on an intent-to-treat basis. Data will be reported as numbers (percentages) or medians (interquartile ranges: 25th to 75th percentiles). Continuous variables will be compared using the Wilcoxon rank sum test and proportion using the Fisher exact test. Death or proven IFI (primary end-point) will be evaluated at end-of-study and analyzed with survival methods of Kaplan-Meier estimate for each treatment group of full analysis set (randomized patients). Patients who received at least one dose of study drug will be kept in the intent-to-treat analysis even if invasive *Candida* infection was diagnosed during the first 48 hours of randomization.

Secondary efficacy end-point will be evaluated similarly in the intent-to-treat population. The Cox model will be used if adjustment variables must be taken into account. Proportional assumption will be assessed using graphical methods. All the analyses will be stratified by center. No interim analyses are planned. Missing, unused data or outliers will lead to queries. In the cases where they are confirmed, the data concerning the independent variables will be replaced using multiple imputation methods.

According to French legislation, serious unexpected adverse events will be immediately communicated to the French Health authorities and placed in the Eudravigilance™ database. Safety analyses will be done after 50 and 150 included patients or more upon request of DSMB. Vital signs, body weight and temperature, clinical examinations and laboratory tests will be described. Adverse events will be summarized by system after coding. Incidence of patients with at least one AE general or of special interest, per group, will be calculated and summarized in tables. The incidence of AEs will be described per group in tables, according to severity and imputability to study drug. SAEs will also be summarized in tables and detailed in a separate section and reported to the Regional Pharmacovigilance Center. Changes between baseline and end-of-treatment in vital signs and laboratory tests will be described for each treatment group.

PK analysis will be performed according to a population approach (NONMEN software, ICON, Dublin, Ireland). PK parameters of population (means, inter and intraindividual variability) will be estimated with overall data. The following covariates will be studied: age, weight, concomitant medications, proteinemia, albuminemia, prothrombin time, ALT, AST, nature and volume of intravenous fluids that were used, evolution of weight between admission to ICU and day of PK sampling, evolution of proteinemia between admission to ICU and day of PK sampling. Significance of relationship between covariates and PK parameters will be evaluated with the modification of the objective function and impact on interindividual variability of PK parameters. A decrease of at least 3.84 points (α = 5%) in objective function will be used as significance criteria for the ascendant phase. For the decreasing phase, an increase in objective function of at least 6.63 points (α = 1%) will be required. The final model will be validated using visual predictive checks and normalized prediction errors. PK/PD statistical analyses will be performed by the Pharmacology Department of Dr V Jullien.

## Discussion

Regarding benefits to expect from empirical or preemptive SAT in critically ill non-immunocompromized patients, the current literature is inconclusive and trials demonstrating the efficacy of SAT in colonized patients with unresolved sepsis and organ dysfunction are warranted.

Appropriate treatment of proven invasive candidiasis needs to be started as early as possible. Indeed, randomized control trials (RCTs) have demonstrated that SAT is effective in cases of proven invasive fungal infection, but these latter represent only 15 to 20% of the SAT prescribed in ICU [12,35]. In high-risk digestive surgery patients managed in ICUs with high annual incidence of candidemia (that is, > 1 to 2%), prophylaxis may be effective. However, regarding the so-called preemptive or empirical therapy, no clear demonstration of efficacy has been published. One before-after study showed decreased ICU-acquired candidemia using a colonization index-based fluconazole therapy, but no survival benefits. A RCT failed to demonstrate an impact of fluconazole on invasive fungal infection or mortality in critically ill patients with unresolved sepsis and risk factors for candidemia. The issue remains uncertain because diagnosis of invasive fungal infection remains a challenge in ICU. In a one-day prevalence study, the intensivists declared 17% of nosocomial infections to be due to *Candida* spp. [36], however, only 99/14,414 patients developed proven candidemia.

*Candida* colonization is a frequent event in ICU patients [12]. The colonization index, validated 20 years ago in long-term surgical ICU patients, has been largely challenged. For instance, the colonization index positive predictive value is less than 9% in the EPCAN study [12]. Furthermore, in medical ICU patients, 39% developed a colonization index of more than 0.5, while, in the same period, no invasive fungal infections were diagnosed [7]. New tools such as (1–3)-β-D-glucan levels provide promising results in ICU population [37,38]. However, (1–3)-β-D-glucan is not specific of candidiasis, is higher than 80 pg/ml in many ICU patients without invasive candidiasis and decreases slowly under effective treatment [39-41].

New antifungal agents are well tolerated and overtreatment might be considered as safe at a patient level. However, new data from US and Europe clearly demonstrate that overuse of antifungal drugs contributes to both the emergence of *Candida* species that are known to be less sensitive to antifungal agents, as well as to increased MICs of sensitive *Candida* species. Recently, Lortholary *et al*. reported that azole derivatives and candin pre-exposure increases the risk of fungemia due to species with higher MICs to corresponding antifungal agents [42]. Pfaller *et al*. found an increase in rates of fluconazole-resistant *Candida glabrata* intermediate or resistant to candins over time from less than 4% between 2000 and 2002 to more than 12% between 2008 and 2010 [43]. Dannaoui *et al*. reported 20 episodes of fungal infections caused by candin-resistant *Candida* spp. that were harboring diverse and new resistance mutations. For 12 patients, initial isolates (low MICs, wild-type FKS gene) and subsequent isolates (after caspofungin treatment, high MIC, FKS mutation) were genetically identical [44]. We also recently described a significant relationship between SAT consumption and MICs of colonizing and infecting fungi in ICU patients [45]. Obviously, SAT should be used by applying the same rules as are applied to other antimicrobial agents and it must be effective and safe for the patient in question as well as for future patients.

Likewise, we are looking forward to having improved diagnostic strategies so to increase sensitivity, specificity and predictive values of available tools, as well as to reduce diagnostic delays.

Until the results from ongoing trials are available, a demonstration of a clinical benefit of treatment of such patients is warranted in order to solve uncertainties around the issue of deciding antifungal treatment in the ICU setting.

## Trial status

The trial is currently recruiting including patients. The inclusion started the 10 July 2012 and the number of included patients is 50. The estimated length of inclusion time is sixteen months.

## Abbreviations

AE: adverse event; ALT: alanine amino transferase; ANSM: Agence Nationale de Sécurité du Médicament; AST: aspartate amino transferase; AUC: area under curve; Cmax: maximum concentration; Cmin: minimum concentration; CPP: Comité de Protection des Personnes; DSMB: Data Safety Monitoring Board; e-CRF: electronic case report form; EOS: end-of-study; EOT: end-of-treatment; IDSA: Infectious Diseases Society of America; IFI: invasive fungal infection; ITT: intent-to-treat; IV: intravenously; MIC: minimal inhibitory concentration; PCR: polymerase chain reaction; PD: pharmacodynamic; PK: pharmacokinetic; RBC: red blood cells; SAE: serious adverse event; SAT: systemic antifungal therapy; SIRS: systemic response inflammatory syndrome; SOFA: sepsis-related organ failure assessment; WBC: white blood cells.

## Competing interests

JFT received lecture fees from Gilead, Pfizer, Merck, Astellas. JFT’s university and research organization received unrestricted research grants from Astellas, Gilead, Merck and Pfizer companies. JFT participated to scientific committee of epidemiological studies organized by Astellas and Merck companies.

MC has received research grants from Pfizer.

JPG received lecture fees from Astellas, Gilead, Merck and Pfizer, and participated in scientific committee of epidemiological studies organized by Astellas and Merck.

VJ is a member of Astellas scientific committee, received lecture fees from Tibotec and BMS and received research grants from Biocodex and Sanofi.

MW received lectures fees from Astellas, Gilead, Merck, Pfizer, Aventis, Cubist and Astra-Zeneca.

No other potential conflict of interest relevant to this article has been reported.

## Authors’ contributions

JFT drafted the manuscript and contributed to the design of the study. MW, VJ, EA, MC, ES and JPG contributed to the design of the study and helped to draft the manuscript. AV, JFT and VJ prepared the statistical analysis and helped to draft the manuscript. All authors read and approved the final manuscript.

## Authors’ information

Participating centers:

CHU Grenoble: Jean François Timsit, Rebecca Hamidfar-Roy, Khadija Hammi, Silvia Calvino-Gunther, Magalie Chautemps, Lenka Stivalovna, Lilia Baki-Kodja, Marie Joyeux-Faure, Muriel Cornet, Danièle Maubon, Hervé Pelloux

CH Aix en Provence: Olivier Baldesi, Sophie Maurisot, Nathalie Brieu

CH Draguignan: Nicolas Bele, Riyad Farhat, Carole Labat, Christian Zumbo

Aurélie Smets

CH Pontoise: Danièle Combaux

CHI André Grégoire, Montreuil: Vincent Daas, Magalie Ciroldi, Frédéric Tacco, Sonia Roos, Karima Dupre, Eliane Benveniste

CHU Beaujon: Catherine Paugam-Burtz, Arnaud Foucrier, Malek Abazid

CHU Besançon: Jean Christophe Navellou, Joelle Fritzsch, Michele Essert, Fréderic Grenouillet, Eric Bardonnet

CHU Bichat, AP-HP: Michel Wolff, Lila Bouadma, Bruno Mourvillier, Romain Sonneville, Sarah Chemam, Sophie Letrou, Arnaud Philippe, Emmanuelle Papy, Christian Chochillon CHU Bordeaux: Didier Gruson, Lucie Estevez, Ghezzoul Bellabes, Olivier Gerbouins, Isabelle Accoberry

CHU Clermont-Ferrand: Bertrand Souweine, Mireille Adda, Sandrine Corny Peccoux, Denis Pons, Natacha Mrozek

CHU Dijon: Pierre Emmanuel Charles, Bruyere Rémi, Hamet Maël, Therese Devaux, Philippe Fagnoni, Frédéric Dalle

CHU Edouard Herriot, Lyon: Bernard Allaouchiche, Christian Guillaume, Charles-Eric Ber, Johanne Prothet, Thomas Rimmelé, Celine Dubien, Soumia Bayarassou, Catherine Jouvene Faure, Anne Millaret, Christine Pivot, Cecile Gerard, Stephane Picot, Francoise Beyerle, Anne-lise Bienvenu

CHU Edouard Herriot, Lyon: Laurent Argaud, Martin Cour, Romain Hernu, Sylvie Coronzier, Anne Millaret, Christine Pivot, Cecile Gerard, Stephane Picot, Francoise Beyerle, Anne-lise Bienvenu

CHU Montpellier: Samir Jaber, Boris Jung, Mathieu Conseil, Coisel Yannael, Belafia Fouad, Albert Prades, Cyril Breuker, Audrey Castet, Natalie Bourgeois

CHU Montpellier: Kadda Klouche, Laurent Amigues, Sonia Machado, Marianne Serveaux, Annie Rodriguez, Cyril Breuker, Audrey Castet, Nathalie Bourgeois

CHU Pitié Salpêtrière: Jean Chastre, Jean-Louis. Trouillet, Fanny Charbonnier, Arnaud Fekkar

CHU Reims: Joël Cousson, Pascal Raclot, Thierry Floch, Pierre Meur, Magda Warchol, Dominique Toubas

CHU Rennes: Jean-Pierre Gangneux

CHU St Etienne: Fabrice Zeni, Darmon Michael, Matthias Pichon, Maud Coudrot, Sebastien Ninet, Eric Diconne, Hanane El Haouari, Julia Mordini, Raberin Helene

CHU St Louis, AP-HP: Benoit Schlemmer, Elie Azoulay, Virginie Lemiale, Nicolas Maziers, Igor Theodose, Isabelle Madelaine Chambrin

CHU Strasbourg: Ferhat Meziani, David Schnell, Xavier Delabranche, Olivier Martinet, Anne Hutt Clauss, Ermanno Canfolfi, Valérie Bru

Hôpital St Joseph, Paris: Benoit Misset, Maité Garrouste-Orgeas, Julien Fournier, Mohamed Cherifi, Marie-Dominique Kitzis, Yaye Senghor

CHI Versailles: Fabrice Bruneel, Sebastien Cavelot, Anne Pattyn, Catherine Palette

Study Monitoring: Khadija Hammi

Biostatistician senior of the OUTCOMEREA Organization: Aurélien Vesin, Stéphane Ruckly.
